# Investigating the effect of bruxism on maxillary arch length and width in children using three-dimensional digital model analysis

**DOI:** 10.1186/s40510-021-00396-y

**Published:** 2022-01-10

**Authors:** Ebru Hazar Bodrumlu, Fethiye Çakmak Özlü, Hakan Yılmaz, Levent Demiriz

**Affiliations:** 1 Department of Pediatric Dentistry, Faculty of Dentistry, Zonguldak Bülent Ecevit University, Zonguldak, Turkey; 2 Department of Orthodontics, Faculty of Dentistry, Ondokuz Mayıs University, Samsun, Turkey; 3 Department of Orthodontics, Faculty of Dentistry, Yeditepe University, İstanbul, Turkey; 4 Specialist in Pediatric Dentistry, PHD, Zonguldak, Turkey

**Keywords:** Bruxism, Children, Digital dentistry, Digital model, Maxillary arch, Three-dimensional imaging

## Abstract

**Background:**

Bruxism is defined as repetitive jaw-muscle activity characterized by the grinding and clenching of teeth. The prevalence of bruxism in children is extensive, and it can cause irregularities in dental arches. The study aimed to investigate the presence of any effects of bruxism on maxillary arch length and width in children using three-dimensional (3D) digital model analysis.

**Method:**

This study evaluated 30 children with bruxism. For every child with bruxism, a case control without bruxism was selected and matched for gender, age, and dentition. Digital models of the patients’ maxilla were obtained with a 3D intraoral scanner, and width and length measurements between the reference points on the maxilla were obtained on the digital models.

**Results:**

The mean age of the study group was 9.13 ± 1.27. Insıgnificance differences were found between females and males within and between groups in terms of maxillary width and length. Insignificant difference was found between the control and study groups when the lengths of 3R-3L, 4R-4L, 5R-5L, 6R-6L, and IP-M were compared (*p* > 0.05).

**Conclusion:**

Based on the study results, there were no differences in the maxillary arch length and width in patients with bruxism and patients without bruxism.

## Introduction

Bruxism is defined as repetitive jaw-muscle activity characterized by the grinding and clenching of teeth. Although current systematic research has focused more on bruxism in adults, there is no complete interdisciplinary coordination of bruxism in children [1]. According to available studies, the prevalence of bruxism has been found to vary from 3.5% to 49.6% in children [1, 2], and sleep bruxism is the type most frequently observed. Although it is suggested that bruxism begins at approximately 4.9 ± 2 years of age, it is disregarded resulting in a lack of dental intervention. The prevalence of bruxism in children is extensive, and it has been proven that bruxism is seen more frequently in young people and children than in adults [2]. Demir et al. [3] reported the prevalence of bruxism as 12.6% in their studies evaluating 965 Turkish children aged 7–19 years.

Bruxism can be considered to be the most destructive parafunctional activity of the stomatognathic system; it causes abnormal tooth wear and damages periodontal tissues, temporomandibular joints, and muscles. Psychological factors, such as depression and stress, are mentioned in the etiology of bruxism [4]. The most common clinical signs and symptoms of bruxism in oral tissues are: irregularities in the dental arches and periodontium, pulp hypersensitivity, dental mobility, fractures in teeth or restorations, tooth surface loss, pain, temporomandibular disorders, masseter muscle hypertrophy, and headache. Other findings that may be associated with bruxism include malocclusions, such as facial asymmetry, inadequate lip closure, mouth breathing, and anterior and posterior crossbite [5]. Moreover, occlusal alterations and deviations in bony anatomy in the orofacial region came to be seen increasingly as primary causal factors for bruxism [6]. Vieira-Andrade et al. [7] and Kataoka et al. [8] performed cross-sectional studies in children and young adults, respectively, and reported a significant association existed between bruxism and crowding. Also, Toyama et al. in 2019 [9] observed the same results in a cohort study.

In addition to all these, tooth surface loss is the most common finding in bruxism. It is suggested that the irregularity and abnormal relationships that will occur due to tooth surface loss in the occlusion may be the cause of malocclusion [10].

Various malocclusions may cause bruxism, and bruxism may even be the main reason for the occurrence of malocclusion [11–13], since the force created by bruxism can result in the movement of teeth. Malocclusion is a developmental disorder that causes functional and esthetic problems in the maxillofacial system. Identifying the factors that cause malocclusion is crucial for providing proper public health services [8].

Malocclusions have been evaluated in the etiology of bruxism, but few studies have evaluated bruxism’s connection to the etiology of malocclusions [7, 9, 14].

In addition, although the effect of bruxism on oral tissues has been investigated in various studies, no studies in the literature have investigated the effects of maxillary width and length on the basis of digital measurements [14]. For this reason, the present study aimed to investigate the effects of bruxism on maxillary arch length and width in children using three-dimensional (3D) imaging and digital measuring.

## Materials and methods

Ethical approval for the present study was obtained from the Ethics Committee of Bulent Ecevit University (protocol number: 2017–68-09/08), and parental consent was obtained for each child. Children with bruxism, ranging in age between 8 and 11, who visited the clinic at the Bulent Ecevit University Faculty of Dentistry, Department of Pediatric Dentistry during a one year period were eligible to participate in this research. For every child with bruxism, a case control without bruxism was selected and matched for gender, age, and dentition.

The inclusion criteria were: systemically healthy, no syndromes, normal facial morphology, mixed dentition, including erupted and contacted permanent first molars, the presence of dental wear without any trauma history, and no oral habits, such as sucking, tongue thrusting, or mouth breathing. Exclusion criteria were: a systemic and/or mental disease, respiratory disease, such as asthma, a syndrome, taking medication that can affect the central nervous system, early loss of tooth in the dental arch, and the presence of dental caries. The sample size was calculated with a confidence of 95% and a statistical power of 80%.

### Procedure for diagnosing bruxism in the study group

In the diagnosis of bruxism, the temporomandibular joint (TMJ) was evaluated in each patient using the same evaluation method described by Bernal and Tsamtsouris [15], including a clinical examination and a patient questionnaire. In addition, the anxiety levels of each patient were obtained using the Conners’ Parents Rating Scales (CPRS) [16].

Based on these evaluations, children who had an anxiety level higher than 0.75, had two or more signs of TMJ disorder, and who met all the American Academy of Sleep Medicine (AASM) [17], criteria for bruxism (Table 1) were included in the bruxism group. The most important source in diagnosing bruxism in children is the information obtained from the parents. The main problem with this method is that the vast majority of children do not sleep with or close to their parents, so parents are not always aware of the situation [1, 2, 17]. Before the baseline of the study, all the parents were asked to sleep in close proximity to their children for 2 weeks to determine the presence of bruxism. All the children in the control group met the AASM criteria, except the first criterion. After the selection of 30 bruxism patients convenient for the inclusion criteria in the present study, 30 control group patients who did not have a habit of bruxism were included for the control group. In the study group, three children had second and first premolars, and four children had permanent canines. In order to improve the reliability of the study findings, case control without bruxism was selected and matched for gender, age, and dentition. Due to both inclusion criteria and case–control matching, a total of 250 children aged 8 to 11 were evaluated. Of those, 60 children were selected for the study. In the present study, all of the patients’ maxillary dental arches displayed mixed dentition, including central and lateral incisors and permanent first molars.

**Table 1:** The bruxism criteria of the American Academy of Sleep Medicine

1. The parents announced the presence of tooth-grinding or tooth-clenching during sleep in their children
2. No medical or mental disorders
3. No other sleep disorders

**Fig. 1:**
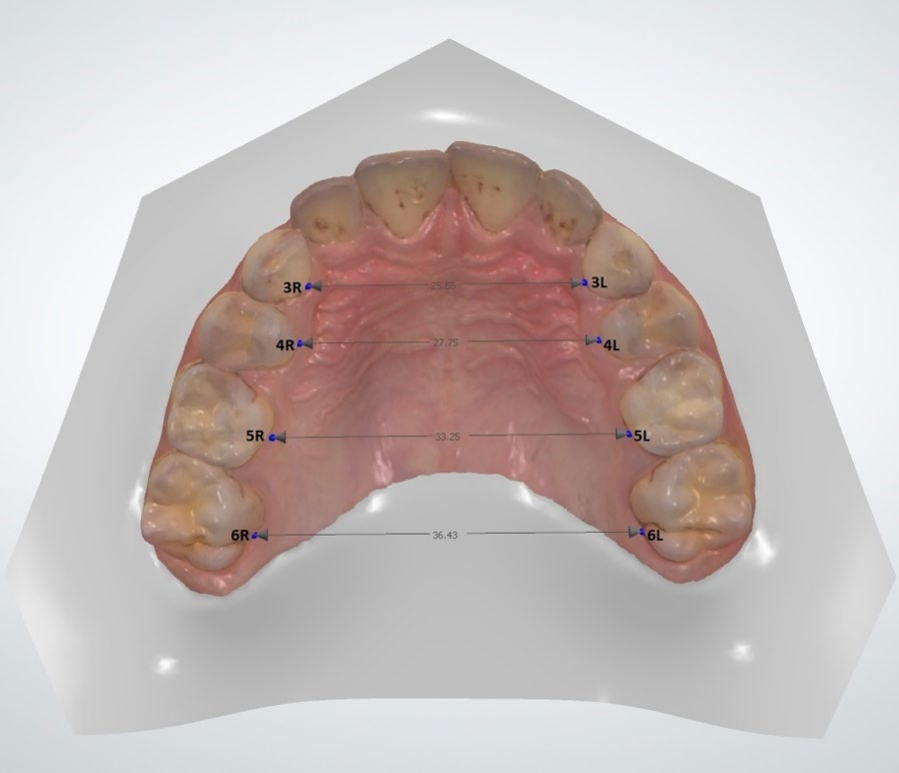
Measuring of the lengths of 3R-3L, 4R-4L, 5R-5L, and 6R-6L on a 3D digital model

**Fig. 2:**
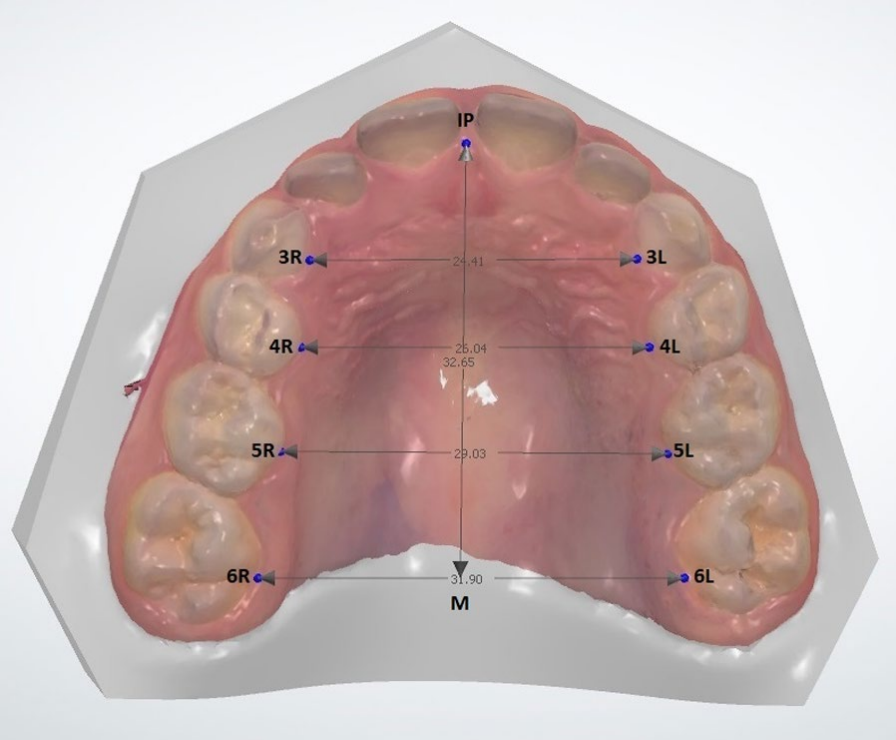
The view of all measurements on a 3D digital model

**Fig. 3:**
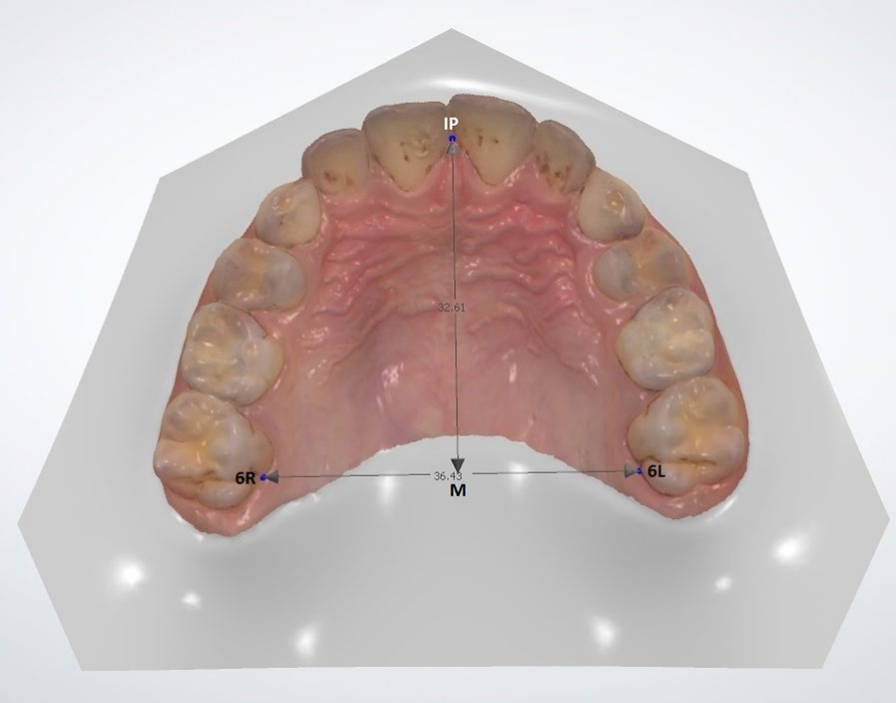
Measuring of the lengths of IP-M on a 3D digital model

**Fig. 4:**
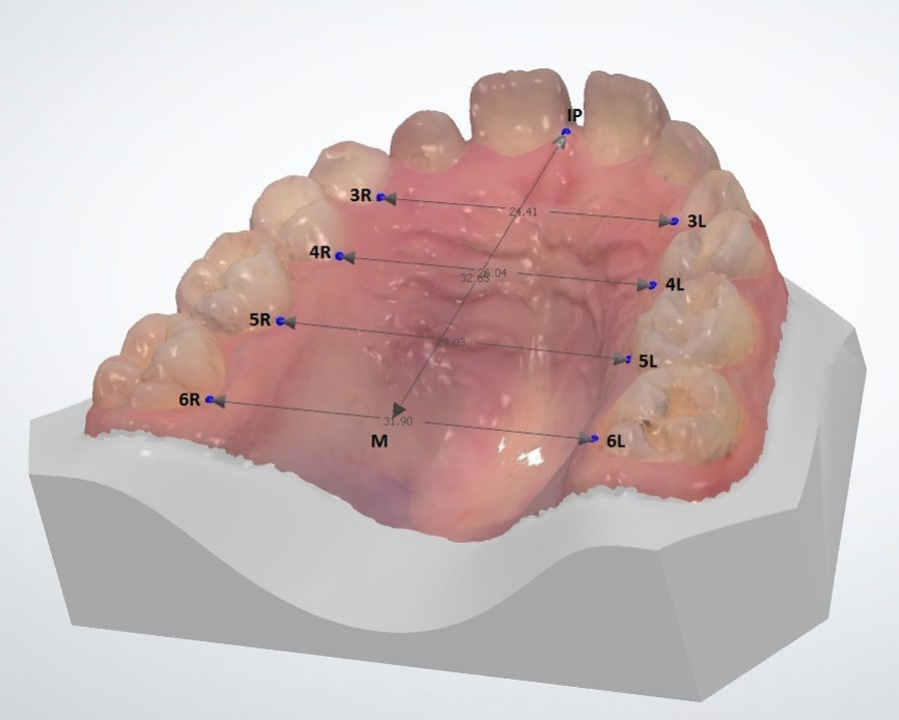
The view of all measurements on a 3D digital model in different side

Following the identification of patients appropriate for the study and control groups, digital models of the maxilla were obtained from the patients using a 3D intraoral scanner (TRIOSColor intraoral scanner, 3Shape, Copenhagen, Denmark). The maxillary arch of each patient was scanned, and the width and length measurements between the reference points on the maxilla were obtained for the digital models using Ortho Analyzer software (3Shape, Copenhagen, Denmark) by one examiner (H.Y.). One examiner performed the scanner (F.Ç.Ö.). One examiner performed statistical analysis (L.D.) and one examiner performed the clinical examination and anamnesis (E.H.B.). The method described by Ferrario et al. [18] was used for the digital measurements. Using this method, the following procedures were done for each patient’s digital model (Figs. 1 and 2):

- 1) The intersections of the palatal sulci of the right and left first permanent molars (sixth teeth, landmarks 6R and 6L), and fifth (landmarks 5R and 5L), fourth (landmarks 4R and 4L), and third teeth (landmarks 3R and 3L) were marked. (Figs. 1 and 2).
- 2) The intersection of the incisive papilla (IP) and the most posterior line of the palatal raphe (RP) was identified and marked.(Fig. 3).
- 3) The line between 6R and 6L and the line perpendicular to 6R-6L starting from the IP were traced; the intersection point was marked as M. (Figs. 3 and 4).

Several points that were nearly equidistant were also marked on each line [19]. After all the markings were completed, the lengths of the all lines were measured in millimeters (mm). In the digital model of 10 patients, the measurements were repeated five times by the same examiner, and the data were statistically analyzed to obtain the reproducibility of the measurements. Based on the analysis, the measuring method was highly reproducible without statistically significant (*p* < 0.05) errors (K: 0.98).

### Sampling size and data analysis

In the present study, sample size were calculated and decided with %95 confidence, 80% Power (power is 1-β; where β is the risk of a type II error of false negative rate, accepted as 0.2 for 80% power) and α = 0.05, accordingly sample statistics of the previously reporting study in Table 1 [20]. Accordingly mentioned study [20] in Table 1, based on the sample statistics of 6R-6L (11.32 ± 1.83) and 5R-5L (13.02 ± 1.73) heights measured for the frontal plane, the sufficient sample size for each group was determined to be at least 30. Therefore, appropriate sample size of present study were determined as 30 units per groups, and totaly 60 units were determined as the total sample size. In addition, the original dataset was categorized by group means to calculate and evaluate with inter-reliability analysis by agreement (Cohen’s kappa coefficient) statistics for the current study. Accordingly, the data set was categorized by evaluating the values below/above the treatment average for each group, assigning 1 to the measurement values below the mean value and 2 to the measurement values above the mean value.

Data were analyzes with using Epi Info 7.1.5 (Licensed to CDC). The descriptive statistics were calculated and summarized as means and theirs standard error of means (Means ± SD). The data were analyzed using a Student’s t-test and logistic regression and Cohen’s kappa coefficient (K) described and detailed in McHugh and Mary (2012) is used to measure inter-rater reliability for observer (or/qualitive ‘categorical’ items). According to Cohen's kappa coefficients, values ≤ 0 as indicating no agreement and 0.01–0.20 as none to slight,0.21–0.40 as fair, 0.41–0.60 as moderate, 0.61–0.80 as substantial, and 0.81–1.00 as almost perfect agreement [21].

## Results

In the study group, 30 children (16 females and 14 males) with bruxism were evaluated. The mean age of the study group was 9.13 ± 1.27. In the control group, 30 children (16 females and 14 males), matched by age, sex, and dentition with the study group, were evaluated. The average age of the control group is similar to the study group. Thus, maxillary arch analysis was performed in 60 patients. In the statistical analyzes, no differences were found between females and males within and between groups in terms of maxillary width and length. No statistically significant difference was found between the control and study groups when the lengths of 3R-3L, 4R-4L, 5R-5L, 6R-6L, and IP-M were compared (*p* > 0.05) (Table 2). There was no statistically significant difference between the groups in terms of measurements when the results were evaluated using logistic regression (*p* = 0.134).

**Table 2:** Length and width measurements of the bruxism and control groups

Measurements	Bruxism (*n* = 30)		Control (*n*= 30)		*p*
	Mean (mm)	SD	Mean (mm)	SD	
3R-3R	24.3287	1.60033	24.6503	1.27830	.393
4R-4R	26.1953	1.25181	27.7113	1.95116	.084
5R-5R	30.5983	1.65073	31.2093	2.03443	.207
6R-6R	34.0943	2.32801	34.3190	2.20752	.703
IP-M	32.7550	1.91595	32.1717	2.12495	.269

## Discussion

The prevalence of bruxism is higher in younger individuals than in older individuals [8, 22]. In addition, in studies evaluating the relationship between malocclusion and bruxism in children, a significant relationship was found between some occlusal factors and bruxism [12, 23, 24]. Ghafournia et al. [11] evaluated the relationship between bruxism and malocclusion and reported a significant relationship between bruxism and primary molars and irritating tooth conditions among preschool children. Regarding occlusal factors, there was statistically significant relationships between mesial step, flush terminal plane, and bruxism. Of the irritating tooth conditions assessed, food impaction, extensive tooth caries, tooth pain, and sharp tooth edges were found to have significant relation-ships with bruxism. In their study evaluating craniofacial morphology and the dental status of bruxist patients, Carra et al. [13] reported that the craniofacial morphology of over 60% of bruxist patients was dental class II, and 28.1% were brachyfacial. These prevalence values were significantly higher than in control subjects (*p* = 0.001 and 0.01, respectively). Bruxist patients showed a lower prevalence of posterior crossbite compared with controls (15.5% vs. 33.8%, respectively; *P* = 0.006). Overall, there were no differences between groups for maximal mouth opening. Compared with controls, bruxist patients were more at risk of experiencing jaw muscle fatigue, headache, and loud breathing during sleep. Pereira et al. [25] stated that there is a direct relationship between posterior crossbite and clinical signs of bruxism.

Bellerive et al. [12] evaluated the possible reduction of bruxism after rapid palatal expansion therapy. They reported a higher percentage of bruxism in children with maxillary transverse deficiency (37%), which is considered to be a risk factor for sleep-disordered breathing [20], and most children with bruxism (65%) reduced their rhythmic masticatory muscle activity episode index after expansion [12]. Bellerive et al. [12] demonstrated that palatal morphology should be evaluated in bruxism. The relationship between bruxism and occlusion has not been well understood although it has been investigated in dentistry [23]. Although some dentists have suggested that malocclusion may be the etiologic factor of bruxism, a recent review concluded that there is no evidence to support that belief [14]. However, bruxism may be a risk factor of malocclusion [7, 9, 13, 14] because the wearing of dental tissues increases since bruxism generates higher forces due to the increased activity of the masticatory muscles, and the contacts between antagonist teeth get larger and flatter than they do in a normal occlusal relationship. This situation allows the horizontal movement of the mandible against the maxilla, which increases stimulation of the alveolar bone [13, 26]. Thus, bruxism may cause some alterations in the dimensions of maxilla, such as leading to a larger palate. Therefore, the present study aimed to investigate the effect of bruxism on maxillary arch length and width.

The alterative effects of bruxism on teeth have also been studied based on facial morphology [27, 28]. However, the effects of bruxism on the shape and/or function of the maxilla have not been sufficiently reported in children. Evaluation of the normal palatal morphology of children with bruxism, in terms of the quantitative analysis of palatal size and shape, has not yet been conducted.

For this reason, the present study evaluated the effect of bruxism on the length and the width of the maxillary arch. The results demonstrate that there were no significant differences between children with bruxism and children without bruxism in terms of arch length and width (*p* > 0.05). Similar to the present study, Nahas-Scocate et al. [29] evaluated bruxism in children during the deciduous dentition period and the presence or absence of posterior crossbite associated with bruxism; they reported that the transverse plane of occlusion was not associated with bruxism. Restrepo et al. [20] evaluated the palatal morphology in bruxist and non-bruxist children; they reported that a child with bruxism may have bigger dental arches than a child without bruxism. They also reported no significant difference in maxillary width, whereas a significant difference was reported in the averages of IP-M measurements. However, in their study, the maxillary dental arches of all the subjects were replicated from a dental plaster model obtained using alginate for the measurements. The models were digitalized, and then the measurements were evaluated on the models. In the present study, measurement data were obtained using 3D-digital models. Restrepo et al. [20] suggested that only 3D computerized analyzes can correctly assessed palatal morphology. In addition, Ferrario et al. [18, 30] highlighted the efficiency of that analysis method. The explanations stated above may explain the difference between our findings and the results reported by Restrrepo et al. [20] Additionally, in the present study more patients were evaluated, so that could be another factor for the differences in the outcome.

Since reports about quantitative analyzes of palatal size and shape in bruxist children are limited, it is difficult to compare the present study’s findings with the results of other studies in the literature. Previous investigations have used either surface-based or landmark-based methods. The present study used the landmark-based method because surface-based methods have some disadvantages, such as taking more time and requiring several scanning processes for each cast. Additionally, the surface-based method is more suitable for evaluating specific patients, such as children with a cleft palate [18, 20, 31].

In orthodontics, digital models are often obtained using an indirect method, which requires transporting the impressions or plaster models to a company for laser or CT scanning [32, 33]. However, this procedure has some risks. Plaster models can fracture [34], and the dental dimensions of the impressions can change [35, 36]. Furthermore, in the intraoral scanning method, patients are not exposed to radiation; this is another advantage when the safety of patients is considered [34]. Based on the advantages stated above, there is high interest in methods that can directly copy the dentition [37].

In recent years, the use of digitized orthodontic records has become more common in clinical practice due to the rapid development of technology. In parallel with this development, using intraoral scanners has become more popular. These devices are used for digital modelling as well as digital model analysis [37]. In the present study, the measurements were also analyzed by using the software on the 3D intraoral scanner device. Abizadeh et al. [32] and Tomassetti et al. [38] reported that measurements were easier to acquire when the digital measuring method was used for the analysis. In accordance with these suggestions, it was reported that there was no significant difference between manual and digital measurements during model analysis [32, 39]. In addition, it was highlighted that measurements on a digital model using software could reduce the rate of error on reference point identification due to the ability to enlarge and clip the images when using a digital model [37, 40]. This helps the examiner easily locate the reference point. However, the identified reference points may vary between examiners, and variations in the reference points may directly affect the reproducibility of the measurements [32, 35]. Therefore, it is important to check the reproducibility of the measurements prior to conducting a study because some degree of interpretation inaccuracy is associated with the measurements regardless of the analysis method that is used [37].

However, many etiological factors play a role in the bruxism, and the morphological effects on the maxillary should be investigated using a larger patient population. The rapid development in technology ensures that the methods used in research are also improving, and the reliability and accuracy of the obtained results may increase. For this reason, further studies are needed to evaluate different morphological characteristics using high reliability methods.

## Conclusion

Based on the results of the study, there were no differences in the maxillary arch length and width in bruxism patients and patients without bruxism. Significant results were obtained with this study, in which technological opportunities were used.

## Data Availability

All data generated or analyzed during this study are included in this article. Further enquiries can be directed to the corresponding author.
